# Faculty Service-Learning Students as Home-Visitors: Outcomes of a Lifestyle Modification Program for Vulnerable Families With Residents in Rural Indonesian Communities

**DOI:** 10.3389/fpubh.2021.597851

**Published:** 2021-05-12

**Authors:** Stephen Wai Hang Kwok, Phyllis Chui Ping Pang, Man Hon Chung, Cynthia Sau Ting Wu

**Affiliations:** School of Nursing, Faculty of Health and Social Sciences, Hong Kong Polytechnic University, Hong Kong, China

**Keywords:** service-learning, home-visit, lifestyle modification, rural Indonesian, diet, exercise, smoking, hand hygiene

## Abstract

**Background:** Risks attributed to chronic diseases, cancer, musculoskeletal discomfort, and infectious diseases among Indonesians were found to be associated with lifestyle behaviors, particularly in rural areas. The aim of this study was to examine the outcomes of a home-visiting lifestyle modification program on improving health risk behaviors among Indonesians living in rural areas.

**Methods:** A total of 160 Indonesians living in rural hamlets in the Yogyakarta Region of Indonesia participated in the program in the period of June 21 to July 21, 2019. In the pre-intervention home interview, learning needs of diet, exercise, hand hygiene, and substance use were identified by using structured assessment tools. In the next home visit, the visitors provided health education and facilitated lifestyle planning based on the related affective and cognitive domains of learning. Subsequent follow-up interviews were conducted 3 weeks after intervention.

**Results:** The results showed that the self-reported intake of vegetables, fruits, meat and salt, cooking with less oil, hand hygiene before eating, number of cigarettes smoked, and symptoms of muscle stiffness significantly improved after the intervention. The lifestyle modification program consisted of the affective and cognitive domains of learning, and could lead to the target behavioral changes in self-reported and observable measures over 1 month.

**Conclusions:** The findings contributed to the framework of community-based health education for health risk reduction and behavioral modification in developing rural communities where health care resources were limited. Further studies with control groups and vigorous objective measures were recommended to elucidate its long-term impacts. The factors leading to its sustainability concerning collaborative care partnerships between community residents and faculty resources are worthy of continued exploration.

## Introduction

Lifestyle behaviors among rural Indonesians have caught research attention related to public health promotion. Indonesians in rural areas were found to consume less vegetables and meat than average, and the prevalence of being overweight was higher than the figures in urban areas. The consumption of processed food which is rich in fat and salt probably contributed to their weight problems (1). Indonesia also had a disproportionately higher consumption of dried processed foods than other low- and middle-income countries in East and Southeast Asia (2, 3). Furthermore, Pujilestari et al. (4) found that rural Indonesians possessed limited awareness of contracting diabetes mellitus. Mboi et al. (5) also reported that dietary risk, tobacco use, high body mass index, high total cholesterol, and low physical activity were highly associated with cardiovascular diseases and diabetes mellitus among Indonesians; whereas, unsafe water, sanitation, and handwashing were primarily associated with infectious diseases.

Smoking and physical inactivity were found to be significant risk factors of non-communicable diseases among Indonesian. Amalia et al. (6) analyzed ~64,000 Indonesians aged 15 or above in the Indonesia Family Life Survey in 2007 and 2014. Compared with 2007, smokers purchased and consumed more cigarettes in 2014. Smoking history was determined to be correlated with cancer, and particularly gene mutations among Indonesians with lung cancer (7). Smoking causes an abnormal protein change and is associated with nasopharyngeal carcinoma (8). In addition, previous studies found that smoking was associated with high morbidity and mortality rates, disease burden, and healthcare cost of cancer; while smoking behavior became more severe than previously found in Indonesia. Kristina et al. (9) determined that the mortality rate and cost of cancer ascribed to smoking was ~31% and USD 1,309 billion, respectively. Moreover, Kristina et al. (10) analyzed the morbidity and mortality of nearly 160,000 cancer cases among Indonesians in the GLOBOCAN 2012 database (11), and ~45,000 cases and 36,000 deaths were related to smoking. In an Indonesian study, Kusmana (12) showed that non-smokers and physical activity were associated with lower risk of mortality and higher survival rate of cardiovascular diseases. Furthermore, being physically inactive and being obese constituted significant risk factors of diabetes mellitus in some investigations (13–15).

Hand hygiene is essential to prevent infectious disease, but not all Indonesians practice hand hygiene before and after critical moments which might contaminate hands and foods. Patunru (16) analyzed data collected by the Indonesian Central Bureau of Statistics (BPS) (17) and the Institute for Economic and Social Research (18) from more than one million Indonesians per year between 1990 and 2015. It was found that, among those who were less likely to practice handwashing before eating or after defecating, the prevalence of diarrhea was higher than among those who had washed their hands. Hirai et al. (19) examined 1,700 Indonesian households interviewed in the Knowledge, Attitude, and Practice (KAP) Survey in 2014 (20). The time before eating was considered a critical moment for hand hygiene which could prevent communicable diseases. However, more than 10% of the respondents were found not to have performed hand hygiene prior to eating.

To promote health behavioral outcomes among rural Indonesian, the conceptual framework guiding the design and implementation of intervention in this study involved targeting affective and cognitive factors. This affective-cognitive-behavioral approach is supported by evidence in the literature. Previous studies demonstrated that affective and cognitive factors exhibited significant correlations with eating behaviors (21, 22) and physical activity (23, 24) among adults, though the psychometric scales used varied across studies. According to Fisher et al. (25), cognitive functioning refers to multiple mental abilities which were quantified using the most basic underlying constructs of abilities such as general intelligence, fluid intelligence, and crystallized intelligence (26–29). Fluid cognitive abilities refer to reasoning, processing, and problem solving which are independent of acquired knowledge. On the other hand, crystallized cognitive abilities refer to acquired knowledge, achievements, or vocabulary, etc. Previous studies found that the need for cognition was positively correlated with knowledge as well as other abilities such as verbal skills (30). In particular, the need for cognition might contribute to knowledge acquisition, learning, and training in older adults (31). Hence, knowledge is not considered completely separate from cognition. As a consequence of the above findings, health education with lifestyle behavioral interventions might focus on recognizing affective factors and fulfilling knowledge needs in the general rural Indonesian population.

## Hypotheses

It was hypothesized that, after the intervention: (1) the intake of vegetables and fruits would increase, and the consumption of salt and artificial seasonings would decrease; (2) cooking with an excessive amount of oil would be less frequent, healthier cooking methods with water and heat would be adopted, and the frequency of practicing hand hygiene prior to eating would increase; (3) the number of cigarettes smoked would decrease; and (4) the frequency and duration of physical exercise would increase, and the symptoms of muscle stiffness would decrease.

## Methods

### Design

A secondary data analysis was conducted for a service-learning program aimed at inducing health-related lifestyle behavioral change among rural Indonesians between June 21 and July 21, 2019. It was a one-group pre and post test design.

### Ethical Approval and Consents

The studies involving human participants were reviewed and approved by the Departmental Research Committee (on behalf of Human Subjects Ethics Sub-Committee) of The Hong Kong Polytechnic University (ID: HSEARS20200708006). We also obtained a letter of consent to access the service evaluation data for the secondary analysis from the University Kristen Duta Wacana. Implied consents were obtained when the participants received the home-visiting services.

### Participants

In total, 160 households were visited. All of the residents were rural Indonesians aged 18 or above. The participants included either adult family members (e.g., husbands, wives, adult children, or household workers) who stayed at home during visits. In addition, they were able to communicate verbally or in writing. The settings were the participants' homes in the three rural hamlets of Pelem, Gowok, and Gebang. The location was the Kebonharjo area of the Yogyakarta Region, Indonesia.

### Home Visitors

Twenty trained faculty service-learning students served as the home-visitors to conduct assessments and intervention under the supervision of two faculty registered nurses and one local social work volunteer. On average, two faculty service-learning students were paired up to visit eight households one-by-one at each time point. In each case, an Indonesian undergraduate served as a translator.

### Assessment Schedule

The home-visit assessments before, immediately after intervention, and at follow-up were, respectively, conducted on June 21–22, June 24–25, and 3 weeks later in mid-July 2019. The first home visit constituted the pre-intervention identification of health risk factors and a physical assessment. The participants completed the interview with a questionnaire assessing their behavioral risks in diet, exercise, hand hygiene, and substance abuse, mainly smoking. They also undertook physical assessments including height, weight, and vital signs including pulse, respiration, and blood pressure measurements. On June 23, the service-learning students visited a local wet market nearby, and examined the variety of foods available and the prices. The aim was to prepare to make recommendations about food choices based on participants' budgets, preferences, and nutritional needs identified in the first visit. In the second home visit, 3 days after the first visit, the participants' habits of diet, physical exercise, and smoking were assessed by completing the self-administered questionnaire. The third home visit was conducted after 3 weeks to evaluate the participants' lifestyle changes again.

### Intervention Framework

The intervention session was conducted in the second home visit. Participants were allowed to review their lifestyle behaviors and related risks identified in the first assessment. Then, health education was provided aiming at reducing health risks through increasing intake of vegetables and fruits, the frequency of hand hygiene before eating, maintaining an adequate range of physical exercise per week, and reducing the consumption of oil, salt, and artificial seasonings, smoking, and symptoms of muscle stiffness. The learning focuses included negative consequences of the risks attributable to the relevant diseases. For instance, critical moments for hand hygiene, such as before eating and after toileting, to prevent communicable diseases were reiterated. Their learning needs, approaches, and strategies were individualized based on the conceptual framework which was the affective-cognitive-behavioral approach.

The affective and cognitive factors of reinforcing the behavioral changes were determined in the intervention session. While addressing the affective factors, the participants were facilitated by the home-visitors to identify psychological barriers in values, beliefs, attitudes, or motivations regarding the behavior modification such as smoking cessation. Family members were encouraged to support the participants to achieve the goals of behavioral change. Regarding cognitive and knowledge factors, the health education was individualized based on the assessment of learning needs concerning “diet,” “physical exercise,” “hand hygiene,” and “smoking.” Throughout the learning process, observation and imitation were required to acquire knowledge and skills. For instance, the stretching exercises were demonstrated by the home-visitors, and participants were tested for key knowledge taught and were requested to return-demonstrate the skills learnt. Participants were also invited to develop personal cues and methods to facilitate behavioral changes.

### Intervention Materials

In the intervention session, relevant standardized learning resources were provided according to the identified learning needs. In addition, standardized equipment was used by the home-visitors, such as one salt bottle (20 or 30 ml) and a 2 g measuring salt spoon, a model egg (as one portion of protein), a standard bowl (250 ml), and a cup (250 ml) for dietary education. Leaflets explaining stretching exercises were distributed to the participants and their families, and a commitment statement was signed by participants who indicated willingness to reduce smoking for achieving behavioral change individually.

### Content Credibility

Educational contents were adopted from materials published by the Hong Kong Centre for Health Protection (32, 33), and recommendations and guidelines developed by the World Health Organization (34–36) and the U.S. Department of Health and Human Services (37, 38). Furthermore, the protocol of stretching exercises was developed based on recommendations from the Guidelines for Exercise Testing and Prescription proposed by the American College of Sports Medicine (39). When encountering medical related scenarios with self-reported hyperthyroidism, hyperuricemia, or urinary stones, the service-learning students consulted supervisors, who were experienced registered nurses, regarding foods not to recommend and/or increase in amount, such as iodine-rich foods (40, 41), purine-rich foods (42, 43), and diets high in salt, purine, and oxalate (44, 45), respectively. In addition, pork was not to be suggested for Muslims due to religious reasons. Furthermore, as alcohol consumption is prohibited among Muslims, exclusion of health promotion on reduction of alcohol use was recommended to service-learning students to demonstrate respect for participants' religious beliefs.

### Outcome Measures

The assessment tools consisted of eight components, which included: (1) personal demographics; (2) education and training received; (3) employment and monthly household income; (4) self-reported health conditions; (5) dietary and hygiene habits; (6) physical exercise habits; (7) smoking habits; and (8) physical assessment results. The personal demographics included age, gender, marital status, religion, and the number of persons in the household. Education and training included the educational background and health-related training received. The employment and monthly household income comprised employment status, work location, working hours per day, working days per week, and average monthly family income.

The self-reported health conditions included perceived health status (1 to 10), happiness (1 to 7), musculoskeletal pain or dysfunction (1 = yes, 2 = no), as well as muscle stiffness at baseline (1 = yes, 2 = no) and at each time point (0–10). Perceived health status, happiness, and muscle stiffness at each time point were measured with a ten-point Likert scale, seven-point smiling faces scale, and eleven-point Likert scale, respectively. A higher score meant better health status, higher level of happiness, and more severe muscle stiffness, respectively.

Concerning dietary habits, the serving of cooked vegetables per day [1 = seldom to 5 = more than one bowl (250 ml = two servings)]; serving of fruits per day (0 = none to 5 = three servings or more); tael of meat per meal [1 = less than one tael (one egg) to 6 = more than five taels]; frequency of using artificial seasoning (e.g., monosodium glutamate) (1 = never to 4 = most of the time); and the amount of salt consumed per day [1 = one salt spoon (2 g) or less to 6 = more than five salt spoons] in the past 7 days were assessed. The cooking practice of deep frying, stir frying, steaming, and boiling in water were questioned on dichotomous scales (1 = yes, 2 = no). The frequency of wiping one's hands before eating (1 = never to 4 = most of the time) were also evaluated.

With respect to physical exercise habits, average minutes spent on physical exercise per day, and days of physical exercise per week (1–7 days) were determined. Doing stretching exercises longer than the recommendation, doing moderate exercises (e.g., walking, cycling, badminton, volleyball) to vigorous exercise (e.g., jogging, running, football, gymnastics) longer than the recommendation; and whether or not working was considered as physical exercise were assessed (1 = yes, 2 = no). Additionally, smoking status at baseline (1 = yes, 2 = no) and the number of cigarettes smoked per day were also evaluated.

The physical assessment included height, weight, body mass index, being overweight (1 = yes, 2 = no), blood pressure, and systolic hypertension status (1 = yes, 2 = no). Measuring tapes and body weight scales were employed to measure the height and weight of the participants, respectively. The cutoff of body mass index defined as overweight was 18.5–23.9 (46). Aneroid sphygmomanometers were used to measure diastolic and systolic blood pressures of the participants while sitting. The threshold of blood pressure defining hypertension was 130/80 mmHg (47–49).

### Statistical Model

In consideration of the differences in demographic variables between hamlets, one-way ANOVA was used for scale variables, and chi-squared test and Fisher exact test were used for categorical variables. Generalized estimating equations (GEE) were used for estimations of the parameters of the targeted outcomes. The probability distribution of the response was assumed to be Gaussian if the outcome was scale-variable, and the scale parameter was estimated with maximum-likelihood. For dichotomous outcomes, the binomial distribution was assumed, and logit link function was used, and the parameters were estimated with a hybrid of the Fisher and Newton-Raphson methods. In this study, the scale variables were the amount of food, artificial seasoning and salt intake, the frequency of hand hygiene, time spent on physical exercise, perceived muscle stiffness, and the number of cigarettes smoked. The dichotomous outcomes were cooking methods used and whether or not stretching exercises and physical exercises were performed for a longer period of time than that recommended. The fixed effects were age, gender, hamlets, and time points. The Wald chi-squared statistics of the model effects were tested with type III analysis. Concerning the repeated measures, the working correlation structure was specified as first-order autoregressive. Robust estimator was utilized to estimate covariance across repeated measures. In addition, the *p*-values of the pairwise comparisons of estimated marginal means were adjusted with the sequential Bonferroni method. The significance level was set at 0.05. IBM SPSS software 25 (IBM, Armonk, NY, U.S.A.) was used for ANOVA, chi-squared, and GEE computations. R 4.0.3 was used for the Fisher exact test.

## Results

### Demographic Characteristics

Table 1 shows the demographic characteristics of the participants. All participants were analyzed. The ages of the participants ranged between 18 and 91 years. Over three-quarters of the participants were married. Moreover, nearly 65% of the participants were Muslims. There were more Catholics in the Pelem hamlet, and more Christians in the Gowok hamlet. On average, there were four people in each household. Approximately 37% of the participants received either primary school education or no schooling. There were more participants with a lower education level in the Gebang hamlet. Furthermore, more than 85% of the participants did not receive health-related training before the intervention.

**Table 1:** Baseline demographic characteristics of the participants.

		**Pelem**	**Gebang**	**Gowok**			
**Variable**	**Range**	**mean (SD)**	**mean (SD)**	**mean (SD)**	**F**		***P***
Age in years	18–91	48.7 (15.5)	52.1 (17.3)	55.5 (18.4)	F_(2,157)_ = 1.85		0.161
Number of persons in the household	1–7	4 (1.4)	4.1 (1.7)	3.8 (1.6)	F_(2,151)_ = 0.64		0.529
Working hours per day	2–19	6.2 (2.8)	7.4 (3.2)	5.6 (2.2)	F_(2,83)_ = 3.10		0.050
Working days per week	2–7	6.2 (1.2)	6.3 (1)	5.7 (1.5)	F_(2,82)_ = 2.22		0.115
Monthly family income in USD (2019)	5.3–354	91.2 (100.6)	43.8 (52.7)	106.9 (96.1)	F_(2,125)_ = 7.50		0.001
Self-perceived health status	1–10	6.8 (1.9)	7.1 (2.6)	6.1 (2.3)	F_(2,154)_ = 2.61		0.077
Happiness	2–7	5.9 (1)	5.5 (1.2)	5.2 (1)	F_(2,156)_ = 4.81		0.009
Height in cm	136–173	156.9 (8)	152.5 (7.8)	155 (7)	F_(2,146)_ = 4.67		0.011
Weight in kg	30–83	55.7 (11.8)	49.6 (10.9)	53.7 (7.6)	F_(2,143)_ = 4.74		0.010
Body mass index	15–34.1	22.5 (4.3)	21.3 (3.9)	22.3 (2.9)	F_(2,142)_ = 1.68		0.191
Systolic blood pressure in mmHg	75–200	125.8 (17.1)	125 (20.1)	131.5 (22.1)	F_(2,156)_ = 1.57		0.211
Diastolic blood pressure in mmHg	50–115	77.5 (11.2)	78.1 (13.3)	77 (8.6)	F_(2,154)_ = 0.13		0.881
**Variable**	**Category**	***n***	***n***	***n***	**Chi-squared**		**Fisher exact**
					**Value**	***p***	***P***
Gender	Male	20	24	19	0.43	0.807	0.840
	Female	28	42	27			
Marital status	Single	7	3	3	6.55	0.365	0.379
	Divorced	0	1	0			
	Married	32	48	35			
	Widowed	4	10	7			
Religion	Christian	0	1	19	137.03	<0.001	<0.001
	Muslim	13	63	27			
	Catholic	35	2	0			
Educational background	Not educated	4	2	4	21.26	0.019	0.005
	Primary school	12	26	10			
	Middle school	13	21	15			
	High school	6	0	8			
	College	9	14	4			
	University	3	2	5			
Health-related training received	Yes	3	9	8	1.98	0.372	0.383
	No	38	55	38			
Employment status	No working experience	3	1	1	20.65	0.024	0.021
	Self-employed	18	30	13			
	Employed	8	11	10			
	Retired	4	2	12			
	Unemployed	1	0	1			
	Housewife	13	18	8			
Work location	Field (farming)	19	32	17	1.30	0.862	0.968
	Health-related	0	1	0			
	Others	4	8	3			
Neck, shoulder, or back pain or dysfunction	Yes	11	8	3	5.58	0.062	0.075
	No	37	58	43			
Knee or leg pain or dysfunction	Yes	4	8	9	2.70	0.259	0.292
	No	44	58	37			
Muscle stiffness in stretching	Yes	39	41	37	7.88	0.019	0.024
	No	7	24	9			
Considering work as physical exercise	Yes	9	13	4	3.08	0.214	0.217
	No	33	52	41			
Smoking in the last 30 days	Yes	13	14	8	1.28	0.526	0.545
	No	35	50	38			
Being overweight	Yes	18	21	13	0.49	0.781	0.793
	No	30	43	20			
Systolic hypertension	Yes	12	18	18	2.55	0.279	0.288
	No	36	47	28			

With respect to employment, ~40% of the participants were self-employed, 25% were housewives, and nearly 20% were employees. There were more retired persons in the Gowok hamlet than in the other hamlets. More than 80% of the participants worked in farming fields, and approximately 17% considered work as physical exercise. The working hours were the longest in the Gebang hamlet, which was over seven hours per day. Concerning family income, the households earned 5 to 354 USD per month. The income in the Gebang hamlet was lower than in the other two hamlets by more than 50%. The income in the Gowok hamlet was the highest (107 USD per month), but the happiness score was the lowest among the hamlets.

Participants in the Gebang hamlet had the best perceived health status. Nonetheless, three-quarters of the participants complained of muscle stiffness when stretching. The Gebang hamlet had a lower prevalence of muscle stiffness than the other hamlets by ~20%. Nearly 13% of the participants complained of pain or dysfunction in the neck, shoulder, or back, and the knee or leg, respectively. The heights of the participants ranged between 1.4 and 1.7 meters, and their weights ranged between 30 and 83 kg. Participants in the Gebang hamlet were shorter in height and lighter in weight than in the other hamlets. Approximately 36% of the participants were defined as overweight. In addition, 30% of the participants had systolic hypertension. Among the 160 participants, there was one self-reported case of hyperthyroidism, one case of urinary stones, one case of gout, and five cases of hyperuricemia. Furthermore, 22% of the participants had smoked in the last 30 days.

### Targeted Outcomes and Estimations

Tables 2, 3 show the regression coefficients of the dependent variables in regard to each independent variable, including age, gender, hamlet, and time point. The amount of cooked vegetables consumed per day (B [95% CI] = −0.3 [−0.5, −0.1]), fresh fruits per day (B [95% CI] = −0.6 [−0.8, −0.3]), and meat per meal (B [95% CI] = −0.4 [−0.6, −0.1]) were significantly lower at pretest than the amount at follow-up. At follow-up, the amount of vegetable intake was nearly two servings per day, which was less than the recommended three servings daily. The amount of fruit intake was approximately one to two servings per day. Moreover, the amount of meat consumed was nearly two taels per meal, which met the requirement when there were three meals per day. The frequency of using artificial seasoning (B [95% CI] = 1.1 [0.9, 1.3]) and the amount of salt consumed per day (B [95% CI] = 0.5 [0.4, 0.7]) were significantly higher at pretest than the intake at follow-up. The amount of salt used decreased to approximately one to two salt spoons (two to four grams) per day, which was less than the upper limit of five grams daily.

**Table 2:** Regression coefficients and estimated marginal means of the amount of food, artificial seasoning, and salt intake.

	**Vegetables per day**	**Fruit per day**	**Meat per meal**	**Artificial seasoning**	**Salt per day**
	**B [95% CI]**	**B [95% CI]**	**B [95% CI]**	**B [95% CI]**	**B [95% CI]**
(Intercept)	4.3 [3.8, 4.7]^*^	3.6 [2.9, 4.4]^*^	1.9 [1.4, 2.4]^*^	2.1 [1.7, 2.6]^*^	1.4 [0.8, 2.1]^*^
Age	0.001 [−0.007, 0.009]	0.005 [−0.008, 0.02]	−0.004 [−0.01, 0.002]	−0.01 [−0.02, −0.003]^*^	−0.003 [−0.01, 0.006]
Male	−0.1 [−0.4, 0.2]	−0.05 [−0.4, 0.3]	0.2 [−0.1, 0.4]	0.1 [−0.1, 0.4]	0.1 [−0.2, 0.4]
Female	0	0	0	0	0
Pelem	0.6 [0.3, 0.9]^*^	−0.5 [−1, 0.02]	−0.1 [−0.4, 0.1]	0.5 [0.2, 0.9]^*^	−0.1 [−0.5, 0.3]
Gebang	0.3 [−0.04, 0.6]	−0.4 [−0.9, 0.05]	0.2 [−0.1, 0.5]	0.5 [0.2, 0.8]^*^	0.05 [−0.3, 0.4]
Gowok	0	0	0	0	0
Pretest	−0.3 [−0.5, −0.1]^*^	−0.6 [−0.8, −0.3]^*^	−0.4 [−0.6, −0.1]^*^	1.1 [0.9, 1.3]^*^	0.5 [0.4, 0.7]^*^
Posttest	0.0005 [−0.2, 0.2]	0.1 [−0.08, 0.3]	−0.08 [−0.2, 0.07]	0.02 [−0.1, 0.2]	0.06 [−0.01, 0.1]
Follow-up	0	0	0	0	0
(Scale)	0.7	1.7	0.7	0.7	1
	**Mean [95% CI]**	**Mean [95% CI]**	**Mean [95% CI]**	**Mean [95% CI]**	**Mean [95% CI]**
Pretest	4.3 [4.1, 4.4]	3 [2.8, 3.2]	1.4 [1.3, 1.6]	3.1 [2.9, 3.3]	1.9 [1.7, 2.1]
Posttest	4.5 [4.4, 4.7]^*^	3.7 [3.5, 3.9]^*^	1.7 [1.5, 1.8]^*^	2 [1.9, 2.2]^*^	1.4 [1.2, 1.6]^*^
Follow-up	4.5 [4.4, 4.7]^*^	3.6 [3.3, 3.8]^*^	1.8 [1.6, 1.9]^*^	2 [1.9, 2.2]^*^	1.3 [1.2, 1.5]^*^

* *p < 0.05*.

**Table 3:** Regression coefficients and estimated marginal means of hand hygiene, cigarettes smoked, time spent on physical exercise, and muscle stiffness.

	**Wiping hands before eating**	**Cigarettes smoked**	**Physical exercise (days)**	**Physical exercise (min)**	**Muscle stiffness**
	**B [95% CI]**	**B [95% CI]**	**B [95% CI]**	**B [95% CI]**	**B [95% CI]**
(Intercept)	4.1 [3.9, 4.4]^*^	11.1 [4.2, 18.1]^*^	4.1 [2.9, 5.3]^*^	17 [4.5, 29.4]^*^	1.2 [−0.2, 2.6]
Age	−0.003 [−0.007, 0.002]	−0.1 [−0.2, −0.02]^*^	0.02 [−0.0001, 0.03]	0.08 [−0.1, 0.3]	0.01 [−0.009, 0.04]
Male	−0.03 [−0.2, 0.1]	−5.5 [−12.3, 1.2]	−0.4 [−0.9, 0.2]	2 [−6.1, 10.1]	0.4 [−0.4, 1.1]
Female	0	0	0	0	0
Pelem	0.01 [−0.2, 0.2]	1.5 [−0.8, 3.8]	0.7 [0.05, 1.4]^*^	−6.5 [−16.9, 4]	0.4 [−0.5, 1.3]
Gebang	−0.03 [−0.2, 0.2]	1.5 [−1.3, 4.4]	−0.6 [−1.3, 0.03]	−2.4 [−10.6, 5.7]	−0.2 [−1.1, 0.7]
Gowok	0	0	0	0	0
Pretest	−0.3 [−0.4, −0.2]^*^	5.6 [2.6, 8.5]^*^	−0.3 [−1, 0.4]	22 [11.7, 32.3]^*^	1.9 [1.5, 2.3]^*^
Posttest	−0.04 [−0.07, 0.004]	2.4 [−0.2, 4.9]	0.5 [0.1, 0.8]^*^	0.9 [−2.5, 4.4]	0.3 [0.02, 0.5]^*^
Follow-up	0	0	0	0	0
(Scale)	0.3	14.3	4.3	754	6.3
	**Mean [95% CI]**	**Mean [95% CI]**	**Mean [95% CI]**	**Mean [95% CI]**	**Mean [95% CI]**
Pretest	3.7 [3.6, 3.8]	9.7 [6.6, 12.8]	4.5 [4, 5.1]	41.3 [31.1, 51.5]	4 [3.6, 4.5]
Posttest	4 [3.9, 4]^*^	6.5 [3.4, 9.7]^*^	5.3 [5, 5.6]^*^	20.2 [17, 23.4]^*^	2.4 [2, 2.8]^*^
Follow-up	4 [3.9, 4]^*^	4.2 [0.3, 8]^*^	4.8 [4.4, 5.2]	19.3 [16, 22.6]^*^	2.1 [1.7, 2.5]^*^

* *p < 0.05*.

Furthermore, the frequency of wiping hands before eating (B [95% CI] = −0.3 [−0.4, −0.2]) was significantly lower at pretest than the frequency at follow-up. At baseline, 94% or more of the participants washed their hands before eating and cooking and after toileting. The proportion of participants who wiped their hands before eating was 84% at pretest. On the other hand, the number of cigarettes smoked per day (B [95% CI] = 5.6 [2.6, 8.5]) was significantly higher at pretest than the number at follow-up. With regard to physical exercise, the number of days of exercise per week (B [95% CI] = 0.5 [0.1, 0.8]) was significantly higher at posttest than the number at follow-up, while the average number of minutes spent on exercise per day (B [95% CI] = 22 [11.7, 32.3]) was significantly higher at pretest than the time spent at follow-up. Moreover, the level of muscle stiffness during stretching (B [95% CI] = 1.9 [1.5, 2.3]) was significantly higher at pretest than the level at follow-up.

Table 4 shows the odds ratios of the dependent variables with respect to each independent variable. The odds of usual cooking by deep frying (OR [95% CI] = 12.7 [2.8, 57.3]) and stir frying (OR [95% CI] = 2.5 [1.4, 4.5]) were higher at pretest than the odds at follow-up. The likelihood of cooking by steaming (OR [95% CI] = 3.6 [1.8, 7.2]) was higher at posttest than the likelihood at follow-up. At pretest, the odds of cooking by boiling water (OR [95% CI] = 0.3 [0.2, 0.6]) were lower than the odds at follow-up. On the other hand, the likelihood of doing moderate to vigorous exercises longer than the recommended minutes per week at pretest (OR [95% CI] = 30.18 [3.91, 232.86]) was higher than the likelihood at follow-up. However, the odds of doing stretching exercises longer than the recommendation (OR [95% CI] = 0.07 [0.03, 0.16]) at pretest were lower than the odds at follow-up. Yet, the likelihood at posttest was higher than the likelihood at follow-up.

**Table 4:** Odds ratios and 95% confidence intervals of cooking methods and stretching and physical exercises longer than recommendations.

	**Deep frying**	**Stir frying**	**Steaming**	**Boiling in water**	**Stretching exercise**	**Moderate to vigorous exercise**
	**OR [95% CI]**	**OR [95% CI]**	**OR [95% CI]**	**OR [95% CI]**	**OR [95% CI]**	**OR [95% CI]**
(Intercept)	0.04 [0.004, 0.41]^*^	0.87 [0.25, 3.01]	0.008 [0.001, 0.12]^*^	3.11 [0.99, 9.8]	4.19 [1.05, 16.7]^*^	0.005 [0, 0.061]^*^
Age	1 [0.97, 1.03]	0.99 [0.97, 1.01]	1.01 [0.99, 1.04]	1 [0.98, 1.02]	1 [0.98, 1.02]	1.01 [0.99, 1.04]
Male	0.66 [0.29, 1.49]	0.92 [0.49, 1.74]	1.25 [0.53, 2.96]	0.76 [0.42, 1.39]	0.6 [0.31, 1.15]	1.53 [0.66, 3.56]
Female	1	1	1	1	1	1
Pelem	1.51 [0.65, 3.48]	0.26 [0.12, 0.55]^*^	2.51 [0.41, 15.47]	1.26 [0.59, 2.68]	0.67 [0.3, 1.5]	2.27 [0.79, 6.52]
Gebang	0.27 [0.1, 0.7]^*^	0.46 [0.23, 0.96]^*^	9.33 [1.87, 46.57]^*^	1.36 [0.66, 2.83]	0.77 [0.36, 1.63]	1.75 [0.63, 4.87]
Gowok	1	1	1	1	1	1
Pretest	12.7 [2.81, 57.3]^*^	2.49 [1.37, 4.53]^*^	1.43 [0.61, 3.33]	0.32 [0.18, 0.58]^*^	0.07 [0.03, 0.16]^*^	30.18 [3.91, 232.86]^*^
Posttest	1.39 [0.2, 9.55]	1.09 [0.63, 1.9]	3.61 [1.81, 7.23]^*^	0.97 [0.58, 1.64]	2 [1.01, 3.96]^*^	2.26 [0.25, 20.11]
Follow-up	1	1	1	1	1	1
(Scale)	1	1	1	1	1	1

* *p < 0.05*.

## Discussion

The following hypotheses were supported by the results of the current study: (1) vegetable, fruit, and meat intake would increase, and the consumption of salt and artificial seasoning would decrease; (2) cooking with a large amount of oil would be less frequent, a healthier cooking method with water and heat (steaming or boiling) would be adopted, and the frequency of practicing hand hygiene before eating would increase; and (3) the number of cigarettes smoked would decrease. The amount of cooked vegetables and fresh fruits consumed per day and meat intake per meal increased after the intervention. Nevertheless, vegetable intake was still less than the recommended number of daily servings even if the intake was increased after the intervention. The intake of artificial seasoning and salt also decreased post-intervention.

Several potential reasons existed for not meeting the recommendations after the intervention. First, low-income might have limited food choice. The average monthly income per capita in Indonesia was USD 183 in 2019 (50), which was even higher than the mean monthly household income of the three rural hamlets in the present study. Second, the level of reluctance to change, including values and attitudes, was neither quantified nor measured; and the ability to retain the knowledge and skills learned among participants was not assessed after the intervention session, despite the fact that the self-reported behavioral change was measured. Additionally, the efficacy of the intervention could not be evaluated based on the assessment results in 1 month if the affective-cognitive-behavioral changes were not assessed. Third, visits within 1 month might not be sufficient for sustainable behavioral change. Indeed, Mitchell et al. (51) studied 26 RCTs of dietary consultations in primary care settings in a systematic review, and found that the duration of interventions commonly last between 3 and 12 months in order to achieve a quality change.

Nevertheless, the intake of vegetables, fruits, and meat significantly increased despite being less than the recommendation; and the consumption of salt and artificial seasonings was significantly reduced after the intervention in the current study. Marcus (21) reported that the significant differences in affects and cognitions between groups of different eating behaviors appeared to persist in spite of a behavioral intervention in an RCT with 6-month and 1-year follow-ups, although there were some risk of bias, such as unclear randomization, allocation concealment, and blinding. Future studies should measure key variables in the behavioral change model adopted, in addition to the targeted outcome, over a longer time period with a control group.

Another positive result in this study was that the likelihood of cooking which required a large amount of oil was reduced. The practice of steaming increased initially, but this was not maintained at the follow-up. The likelihood of usual cooking by boiling in water rose at post-intervention and then further at follow-up. It seems that boiling in water was the preferred healthy cooking method among the participants. In addition, cooking method might depend on the types of food chosen. For example, vegetables and meat were usually cooked in boiling water, while fish was often cooked by steaming. With regard to hand hygiene, the frequency of wiping hands before eating increased post-intervention. Indeed, the percentages of participants who would wash their hands before eating and cooking and after toileting were high at baseline. The percentage of those who would wipe their hands before eating was also high at pretest. Overall, the results in the current study showed improvement in hand hygiene, which is essential for the population to prevent the transmission of infectious diseases.

Regarding smoking habit, the self-reported number of cigarettes smoked fell after the intervention. Black et al. (52) conducted a systematic review and meta-regression of RCTs of behavioral change interventions for biochemically verified smoking cessation at 6 months or longer. Higher cessation rates were found to be predicted by behavioral cues, self-regulation, commitment, reward, and identity associated with the changed behavior. Furthermore, nicotine dependence, mental health status, and mode of intervention had moderating effects. In the current study, the participants were invited to formulate their own plan and cues for smoking cessation/reduction from affective, cognitive, and behavioral perspectives. For example, they were advised to use the money saved after quitting/reducing smoking as a reward to purchase a gift for themselves. Moreover, family members were encouraged to offer a reward for any successful changes.

The other potential factors of smoking cessation identified by Black et al. (52) were not measured in the current study. Moreover, the intervention session only lasted ~1 h, and the follow-up period was only 3 weeks. In addition, the reduction in the number of cigarettes smoked was not biochemically verified. The results of the current study, however, showed that an intervention session of 1 h focusing on cues and rewards was able to significantly reduce the self-reported number of cigarettes smoked after 3 weeks among rural Indonesian adults, although a control group was absent. In future studies, experiments with a control group, for example RCT, should be conducted to examine other potential factors and objective outcomes of smoking cessation with longer follow-up, particularly for those who live in rural areas.

The hypothesis that the frequency and duration of physical exercise would increase was not supported, despite the decrease in symptoms of muscle stiffness. Concerning physical exercise, the frequency of physical exercise improved at posttest, but this was not sustained. The duration of exercise also decreased, irrespective of the change in the frequency of exercise. In this study, the frequency and duration of physical exercise included both stretching exercises and moderate to vigorous exercise. Specifically, the likelihood of performing moderate to vigorous exercise longer than the recommendation decreased after the intervention. On the other hand, the odds of doing stretching exercises longer than the recommendation increased post-intervention.

There were several probable reasons for participants to attempt stretching exercises instead of moderate to vigorous exercise, as reported by the Indonesians living in rural areas. First, a majority of the participants were farmers who worked 6–7 h per day farming in the fields, and approximately one-sixth of them thought that their work constituted physical exercise. Second, those who complained of muscle stiffness, musculoskeletal pain, or dysfunction might find the stretching exercises a superior option for their symptoms. The results of the current study showed that three-quarters of the participants complained of muscle stiffness when stretching. In hamlets which had a higher prevalence of muscle stiffness than the others, such as the Pelem and Gowok hamlets, the participants had a poorer perceived health status. In addition, more than one-eighth of the participants had pain or dysfunction in the neck, shoulder, or back, and the knee or leg, respectively.

Previously, Udom et al. (53) studied 450 Thai rubber farmers in a cross-sectional survey. The prevalence of low back pain at baseline and after 12 months were 33 and 56%, respectively. Later, Rachmi et al. (54) examined 117 Indonesian dairy farmers in a cross-sectional study in 2017. They found that the prevalence of knee pain was 88%, and this may be associated with the squatting position and the years of work. In the present study, the participants might adopt the stretching exercises learned in the intervention session to relieve their musculoskeletal symptoms, and significant self-reported improvement was evidenced in the results. Experiments with a control group and random group allocation, however, are recommended in future investigations.

## Conclusion

The home-visiting lifestyle modification program addressing the interlink of affective, cognitive, and behavioral needs was found to make a significant difference in modifying lifestyle behaviors and musculoskeletal symptoms over 1 month among Indonesian adults living in rural areas. Further studies with randomized controlled trials focusing on specific health risk behaviors and symptoms are required. Investigations of the generalizability of the results and the long-term impacts in similar socioeconomic and cultural contexts are also warranted.

## Data Availability Statement

The raw data supporting the conclusions of this article will be made available by the authors upon reasonable request.

## Ethics Statement

The studies involving human participants were reviewed and approved by Departmental Research Committee (on behalf of Human Subjects Ethics Sub-Committee), The Hong Kong Polytechnic University (ID: HSEARS20200708006). Implied consents were obtained when the participants received the home-visiting services.

## Author Contributions

CW and PP conceived the idea of the service program. PP, MC, and the undergraduates implemented the intervention and collected the data. SK completed the data analysis and drafted the manuscript. All authors contributed to the article and approved the submitted version.

## Conflict of Interest

The authors declare that the research was conducted in the absence of any commercial or financial relationships that could be construed as a potential conflict of interest.
